# Binary Adsorption Equilibria of Three CO_2_+CH_4_ Mixtures on NIST Reference Zeolite Y (RM 8850) at Temperatures from 298 to 353 K and Pressures up to 3 MPa

**DOI:** 10.1021/acs.jced.4c00358

**Published:** 2024-10-03

**Authors:** Carsten Wedler, Alvaro Ferre, Hassan Azzan, David Danaci, Camille Petit, Ronny Pini

**Affiliations:** † Department of Chemical Engineering, Imperial College London, SW7 2AZ London, United Kingdom; ‡ Laboratory of Chemical Process Engineering, Technical University of Munich, 94315 Straubing, Germany; § The Sargent Centre for Process Systems Engineering, Imperial College London, SW7 2AZ London, United Kingdom; ∥ I-X Centre for AI in Science, Imperial College London, W12 0BZ London, United Kingdom

## Abstract

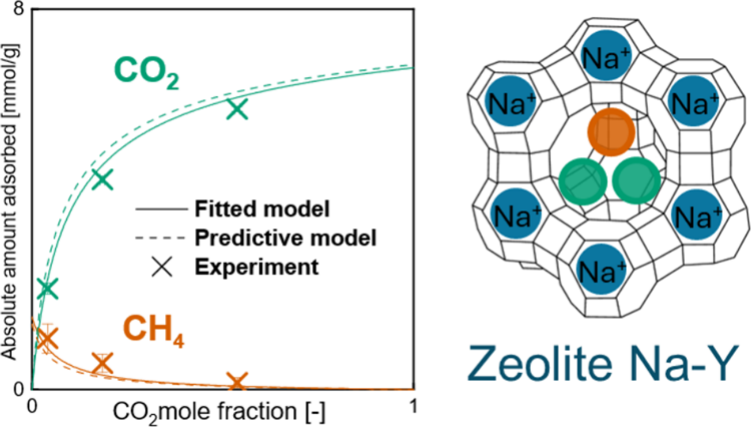

Adsorption equilibria of CO_2_, CH_4_, and their mixtures were measured on binderless pellets of NIST reference zeolite NaY (RM 8850) using a static gravimetric setup. The unary adsorption isotherms are reported at temperatures from 298 to 393 K up to a pressure of 3 MPa and compare favorably with independent results on RM 8850 powder. The competitive adsorption measurements were performed at temperatures from 298 to 353 K and up to 3 MPa for three premixed gas mixtures with CO_2_ molar feed compositions of 0.25, 0.50, and 0.75. The results constitute the first competitive adsorption dataset reported for any of the NIST reference materials. RM 8850 shows a strong selectivity for CO_2_ adsorption toward CH_4_. The experimental unary and binary adsorption isotherms are accurately modeled using the simplified statistical isotherm model (SSI). Notably, the agreement with the model improves only slightly (and within experimental uncertainties) when the whole dataset is used for parameter fitting as opposed to only using the unary data.

## 1 Introduction

The suitability of adsorbents for gas separation or storage applications largely depends on the equilibrium adsorption properties of the adsorbent–adsorbate(s) system. Specifically, the single- and multicomponent equilibrium adsorption isotherms of the species of interest incorporate useful information to rationalize process-level performance of the selected adsorbent.^1−3^ While adsorption equilibrium data of unary gases close to atmospheric pressure are rather common, measurements at elevated pressures are less frequent, more challenging to conduct, and often exhibit poor reproducibility. These difficulties have emerged thanks to several initiatives, which report and compare unary adsorption isotherms measurements of industrial gases, e.g., CO_2_, CH_4_, C_2_H_6_, and H_2_, on both natural and commercial adsorbents obtained in different laboratories using different techniques.^4−12^ A notable effort is currently led by the US National Institute of Standards and Technology (NIST), which has initiated interlaboratory studies on the adsorption of different unary gases at elevated pressures on so-called reference materials, namely: a high-pressure adsorption isotherm of CO_2_ on ammonium zeolite ZSM-5 (RM 8852)^13^ and a high-pressure isotherm of CH_4_ on zeolite NaY (RM 8850).^14^ To this end, NIST provides the adsorbents themselves and standardized protocols to all laboratories involved in the study. Key elements of the experimental protocol include: activation temperature and time; evacuation using a turbomolecular pump; purity of the adsorptive gas of no less than 99.999% (as determined by the supplier); and the measurements of two repeated isotherms for two separate aliquots. In these and previous interlaboratory studies, when discrepancies occurred, these were mostly due to incomplete sample activation, inaccurate determination of sample mass and volume, flawed operating equations and/or incorrect equation of state of the adsorptive gas. Together, these data sets on reference materials provide research laboratories in industry and academia focal points to validate their adsorption measurements, representing a key step toward greater reproducibility of adsorption studies.

Yet, in many industrial applications, adsorption is a competitive process of gas mixtures and for a reliable process design, the competitive adsorption properties should be studied as well (see for example^15−24^). However, a comprehensive review of gas mixture adsorption data by Cai et al. has shown that competitive adsorption data is still scarce.^25^ Due to the inherent complexity of measurements with gas mixtures,^26−28^ their reproducibility still represents a major challenge in adsorption sciences. To the authors’ best knowledge, experimental competitive adsorption data on NIST reference materials has not been reported in the literature yet. To address this gap, we have investigated unary and binary adsorption of CO_2_, CH_4_, and three CO_2_+CH_4_ mixtures on NIST reference zeolite NaY (RM 8850) pellets covering temperature and pressure ranges of 298 to 393 K and 0.03 to 3 MPa, respectively. Our study builds upon the previous work by NIST on CH_4_ adsorption on the same reference zeolite. We have rigorously analyzed the experimental uncertainty by following the procedure of the “Guide to the expression of uncertainty in measurement” (GUM)^29^ as well as by Monte Carlo uncertainty propagation. This data set is of particular interest for the separation of CO_2_ from CH_4_-rich gases, relevant for natural gas or biogas upgrading. Indeed, depending on process configuration, separation pressures for these applications can vary from vacuum to pressures up to 3 MPa.^30,31^ We have described the unary and binary adsorption data using the simplified statistical isotherm model by Ruthven,^32^ including its formulation for binary gas mixtures, also developed by Ruthven et al.^33,34^ In applying the model, we have adopted and compared quantitatively two different approaches. In the first one, the model is fitted to the unary data and is applied to the binary data in a predictive fashion. In the second one, the model is parametrized using the whole data set. Monte Carlo simulations for uncertainty propagation were used to estimate the uncertainties of the obtained model parameters for both approaches.

## 2 Materials and Methods

### 2.1 Materials

For this study, we used the NIST reference zeolite RM 8850, which is a faujasite (FAU) zeolite with a unit cell formula for the aluminosilicate framework of Na_54.0_[Al_54.1_Si_137.9_O_384_]·245.1H_2_O.^35^ The 245.1 represents the number of water molecules that could be entrapped within one unit cell of the aluminosilicate framework.^36^ We used the structural parameters of this material as reported by Nguyen et al.^37^ the skeletal density as ρ_s_ = (2.523 ± 0.014) g/cm^3^ and the micropore volume as ν_mi_ = (0.358 ± 0.003) cm^3^/g. RM 8850 was supplied by NIST as a fine powder. However, for this study, we prepared cylindrical pellets with a diameter of about 5 mm, a length of (3–4) mm, and a weight of (70–80) mg. The pellets were formed without the addition of any binder or other materials using a hydraulic pellet press (Manual Hydraulic Press, Specac Ltd.) with a tonnage of 0.4 t for 30 s, which corresponds to a pelletization pressure of 0.2 GPa. We performed N_2_ physisorption measurements at 77 K using a commercial volumetric instrument (Quantachrome Instruments, type Autosorb iQ) at relative pressures from 5 × 10^–7^ to 0.99. The sample was degassed ex-situ for 16 h at 623 K. The pore volume was calculated by fitting the nonlocal density functional theory (NLDFT) kernel for zeolites/silicas with cylindrical/spherical pores as implemented in the commercial software ASiQwin to the measured N_2_ equilibrium data. We obtained a micropore volume for the pellet sample of 0.359 g/cm^3^, which agrees with independent estimates for a powder sample.^37^ We have additionally compared unary adsorption isotherms measured on powder and pelletized samples, as described in Section 3.1.

For the unary adsorption measurements, CO_2_ and CH_4_ were used as described in Table 1, including the purity of the gas in terms of the mole fraction *y* [−]. The binary adsorption measurements were conducted with three CO_2_+CH_4_ mixtures with nominal CO_2_ mole fractions of 0.25, 0.50, and 0.75 as described in Table 2. The supplier of these cylinders reports that the standard uncertainty *u* of the composition depends on the methane mole fraction, *u*(*y*) = 1% × *y*_CH4_. Table 2 also reports the compositions of the three mixtures which we have estimated by comparing the mass density of the mixture (measured using the gravimetric instrument used for the adsorption studies – Section 2.2) with values obtained upon application of a suitable equation of state. Details on this procedure can be found in Section 1 of the Supporting Information (SI S1). The mole fractions determined using this approach were used for the analysis of the binary adsorption data.

**Table 1:** Description of the Materials Used for the Adsorption Measurements Where *y* Denotes the Mole Fraction

material	CAS number	supplier	purity *y*
carbon dioxide (CO_2_)	124-38-9	BOC	0.99995
methane (CH_4_)	74-82-8	BOC	0.995
nitrogen (N_2_)	7727-37-9	BOC	0.999992
zeolite NaY (RM 8850)	1318-02-1	NIST	

**Table 2:** Nominal Mole Fraction *y*_f_ of the Fed CO_2_+CH_4_ Gas Mixtures as Stated by the Supplier (sup) and Determined via a Comparison of Density Measurements with an Equation of State (EOS)^a^

	supplier	*y*_f,CO2,sup_	*y*_f,CH4,sup_	*y*_f,CO2,EOS_	*y*_f,CH4,EOS_
mixture 1	BOC	0.25	0.75	0.248	0.752
mixture 2	BOC	0.50	0.50	0.492	0.508
mixture 3	BOC	0.75	0.25	0.748	0.252

a For the data analysis, the values obtained from the comparison of density measurements with the EOS were used and are in the following referred to as *y*_f,*i*,EOS_ = *y*_*f*,*i*._

### 2.2 Gravimetric Adsorption Measurements

**Figure 1:**
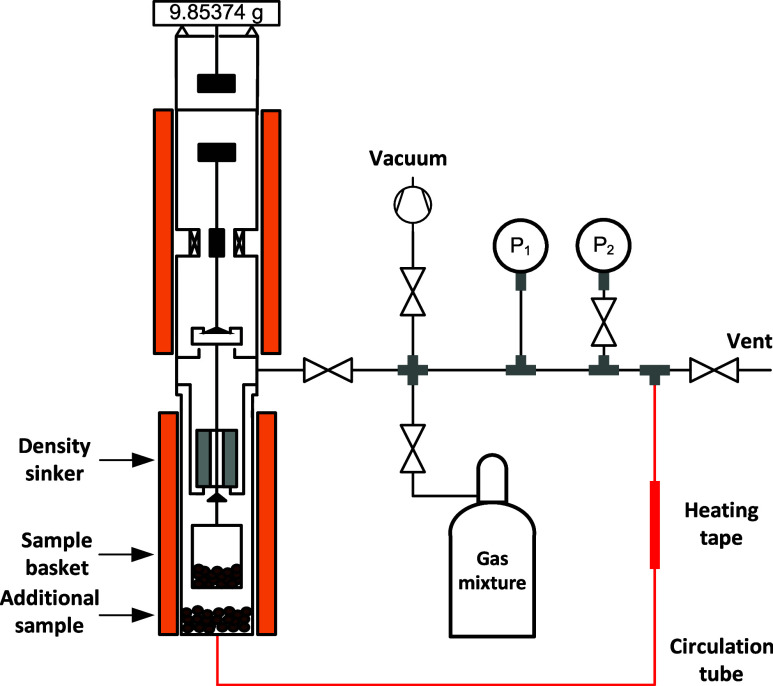
Gravimetric adsorption system used for the adsorption measurement. For the binary measurements, an additional adsorbent sample was added to the bottom of the measurement cell, and the heating tape was constantly heated at 353 K to enable circulation of the bulk gas driven by a density gradient. The system is housed in a laboratory equipped with temperature control (ambient temperature, *T* = 294 ± 2 K).

A magnetic suspension balance (Rubotherm, since 2016 TA Instruments, type ISO-SORP) was used to measure the unary and binary adsorption isotherms. This setup, shown in Figure 1 and employed for previous studies on unary adsorption,^38,39^ was modified to enable the binary adsorption experiments. Using the static gravimetric method, the adsorption-induced compositional shift was determined from the change in gas phase density. A larger mass of adsorbent leads to a stronger shift in composition, resulting in a lower uncertainty of the experimental data. However, as the amount of sample that could be filled into the sample basket was limited, an additional mass of sample was instead added to the bottom of the measurement cell. Due to the compositional change, we had to ensure that the gas phase was homogeneously mixed. As described by Yang et al.,^23^ appropriate mixing can be achieved by installing a heated circulation tube. By fitting this circulation tube equipped with heated tape (marked red in Figure 1), we were able to ensure a homogeneous gas phase. We note that (i) without this tube, we obtained inconsistent results, including negative adsorption loading values, and (ii) the circulation tube had no measurable effect on the stability of the balance reading or temperature.

#### 2.2.1 Unary Adsorption Measurements

Unary adsorption experiments were conducted with CO_2_ and CH_4_ at four temperatures *T* = (298, 333, 353, and 393 K) and pressures *p* = (0.03 to 3) MPa. The RM 8850 pellets were filled into the measurement basket and before each isothermal measurement, the sample was degassed in situ at a temperature of 633 K for 16 h. After cooling down to measurement temperature, the sample mass *m*_s,basket_ [g] in the basket was determined in vacuum as 0.79740 g. This value was repeated for several isothermal measurements and used to calculate the volume of the sample *V*_s,basket_ [cm^3^] by considering the reported skeletal density ρ_s_ [g/cm^3^] for RM 8850.^37^

The excess adsorbed mass *m*_ex_ [g] was determined according to eqs 1–3, where *m*_met_ [g] is the mass of all suspended metal parts, *V*_met_ [cm^3^] is the volume of the suspended metal parts, and *V*_sk_ [cm^3^] is the volume of the titanium sinker. The values for *m*_met_ and *V*_met_ have been determined from previous high-pressure buoyancy measurements within the magnetic suspension balance and for *V*_sk_ the value was determined by a certified calibration laboratory. All three values are reported in the SI S3. In eq 1, ρ_gas,exp_ [g/cm^3^] is the bulk density of the adsorbate that is measured experimentally using the calibrated density sinker, as given by eq 4. *W*_0_ [g], *W*_1_ [g], and *W*_2_ [g] are the measured weights of the suspended parts at the zero point (ZP), measurement point 1 (MP1, only the sample basket is lifted) and measurement point 2 (MP2, sample basket and density sinker are lifted), respectively.
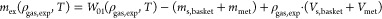
1
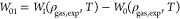
2
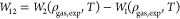
3
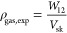
4

The excess adsorbed mass was converted to the molar excess adsorbed loading *q*_ex_ [mmol/g] by considering the molar mass of the gas *M*_*i*_ [g/mol] according to
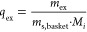
5

Up to this point, we have calculated the adsorption loadings in terms of the excess amount adsorbed,^40^ i.e., the amount of gas present in the system in excess of the amount that would be present if the accessible volume (including the pores of the adsorbent) were occupied by the adsorptive gas in its bulk state. This definition of adsorption uses for the buoyancy force correction the skeletal volume of the adsorbent *V*_s,basket_. Because the latter might be difficult to determine,^41^ other definitions have been proposed, such as the net amount adsorbed,^42^ i.e., the amount of gas present in the system in excess of the amount of bulk gas that would be present in the same system devoid of adsorbent. Yet, for modeling purposes, including the design of adsorption units, the absolute amount adsorbed is needed. To this end, we converted the excess to the absolute adsorbed loading *q*_abs_ [mmol/g] according to eq 6 by assuming a constant volume of the adsorbed phase, for which we took the value of the reported micropore volume ν_mi_ [cm^3^/g] of the adsorbent. A more detailed discussion on the differences between excess, absolute, and net adsorption can be found elsewhere.^43,44^
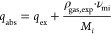
6

#### 2.2.2 Binary Adsorption Measurements

The binary adsorption measurements were conducted with three CO_2_+CH_4_ mixtures (see Table 2) at pressures from 0.1 to 3 MPa at 298 and 333 K and from 0.1 to 2 MPa at 313 and 353 K. During all binary adsorption experiments, the circulation line was constantly heated to a temperature of 353 K and additional adsorbent sample was added to the basket (value reported in S3) and at the bottom of the measuring cell, *m*_s,bottom_, to enable precise determination of the equilibrium composition of the bulk gas. The same equations were used as for the unary adsorption experiments, eqs 1–4, to estimate the excess adsorbed mass of the gas mixture on the sample in the basket *m*_ex,basket_ (called *m*_ex_ in the unary case) and the density of the bulk gas ρ_gas,exp_. To determine the excess adsorbed mass of each component in the gas mixture, the mass balance of the amount of gas filled into the cell has to be established, namely:
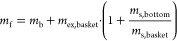
7
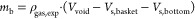
8where *m*_f_ [g] is the total mass of gas fed to the system, which must be equal to the sum of the total excess mass adsorbed on both samples (loaded in the basket and on the bottom of the cell) and the mass of bulk gas *m*_b_ [g] in the system at equilibrium. The latter requires information on the total void volume of the system (without adsorbent present) *V*_void_ [g], which was determined from a preceding calibration experiment described in the SI S2. The mass of adsorbent *m*_s,bottom_ [g] was calculated as follows:
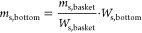
9where *W*_s,basket_ [g] and *W*_s,bottom_ [g] are the mass of the samples determined at atmospheric lab conditions using an analytical balance prior to in situ degassing at 633 K for 16 h and are reported in SI S3. The mass of each component of the gas mixture fed to the system *m*_f,*i*_ [g] is then obtained according to eq 10. As the mole fractions *y*_f,*i*_ of the feed gas (see Table 2) were known, the mass fractions of the feed gas *w*_f,*i*_ [–] were calculated using the molar mass *M*_*i*_ of the individual components according to eq 11.
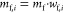
10
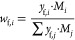
11

Due to the adsorption in the static setup, the equilibrium mole fractions *y*_eq,*i*_ of the bulk phase differed from the initial composition. To calculate the equilibrium mole fractions, the GERG-2008 EOS^45^ was applied as implemented in REFPROP 10.0,^46^ considering the measured density of the gas phase at known temperature and pressure. The mass fractions in equilibrium *w*_eq,*i*_ were calculated according to eq 13 and considered in eq 14 to obtain the masses of the individual gas components in the bulk phase at equilibrium conditions *m*_b,*i*_.
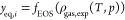
12
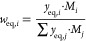
13
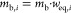
14

Therefore, the excess mass adsorbed of each component *m*_ex,*i*_ was calculated as the difference of mass between that fed into the cell and that present in the gas phase at equilibrium:
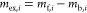
15

The absolute molar adsorbed loading *q*_abs,*i*_ [mmol/g] of each component in the mixture is obtained in an analogous manner as for the unary experiments:
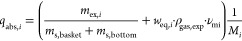
16

The mole fraction of the adsorbed phase *x*_*i*_ [–] is obtained as follows
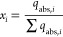
17

### 2.3 Experimental Uncertainty Estimation

The uncertainties of the unary adsorption data were estimated according to the ISO/IEC Guide 98–3:2008 “Guide of the Expression of Uncertainty of Measurements” (GUM),^29^ as suggested by Yang et al.^47^ and applied in adsorption studies using magnetic suspension balances.^23,48^ The combined standard uncertainties *u*_c_ [mmol/g] of the excess and absolute adsorbed amount were estimated individually according to eq 18, where *q* can represent either absolute or excess amount adsorbed. For both uncertainties, the standard uncertainties *u* of the weighing values *W*_01_ and *W*_12_, the sample mass *m*_s,basket_, the skeletal density ρ_s_, and the volume of the calibrated sinker *V*_sk_ were considered. The individual standard uncertainty values contributing to the combined uncertainty of the adsorbed amount are reported in Table 3. We note that for the unary case, the uncertainty of the pressure and temperature measurement do not influence the uncertainty of the adsorbed amount since the measured temperature and pressure values are not used in the calculation of the adsorbed amount. As the uncertainty of the considered pore volume reported by Nguyen et al.^37^ is rather small, no noticeable effect of this value on the uncertainty of the absolute adsorbed amount was found. It was therefore neglected. A sensitivity analysis of each individual contribution to the adsorbed amount led to the sensitivity coefficients ∂*q*/∂*x*. The combined expanded uncertainty was obtained as *U*_c_(*q*) = *u*_c_(*q*) × *k* with a coverage factor of *k* = 2.
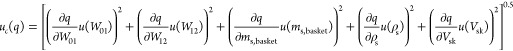
18

**Table 3:** GUM Uncertainty Budget for the Absolute Amount of CH_4_ Adsorbed on RM 8850 at a Pressure of *p* = 1 MPa and *T* = 333 K with Mixture 1^a^

quantity	value	standard uncertainty *u*(*x*)	sensitivity coefficient ∂*q*/∂*x*	uncertainty contribution *u*(*x*) × ∂*q*/∂*x*
*W*_01_	9.18586 g	0.00006 g	560.96 mmol/g^2^	0.0194 mmol/g
*W*_12_	28.77799 g	0.00006 g	–535.96 mmol/g^2^	–0.0186 mmol/g
*m*_s,basket_	1.30182 g	0.001 g	–27.853 mmol/g^2^	–0.0161 mmol/g
*m*_s,bottom_	3.83429 g	0.001 g	0.778 mmol/g^2^	0.0004 mmol/g
ρ_s_	2.523 g/cm^3^	0.014 g/cm^3^	–0.053 mmol·cm^3^/g^2^	–0.0004 mmol/g
ρ_EOS_	0.00678 g/cm^3^	0.1%·ρ_EOS_	2338.8 mmol·cm^3^/g^2^	0.0092 mmol/g
*V*_sk_	4.364 cm^3^	0.002 cm^3^	–3.633 mmol/(g·cm^3^)	–0.0042 mmol/g
*V*_void_	175.48 cm^3^	1 cm^3^	–0.023 mmol/(g·cm^3^)	–0.0131 mmol/g
*T*	333.05 K	0.5 K	0.061 mmol/(g·K)	0.0177 mmol/g
*p*	1.008 MPa	0.002 MPa	–0.195 mmol/(g·MPa)	–0.0225 mmol/g
*y*_f,CH4_	0.752	1%·*y*_f,CH4_	–31.629 mmol/g	–0.1370 mmol/g

a Combined expanded uncertainty (*k* = 2) of *c*: 0.289 mmol/g.

The uncertainties of the binary measurement were also assessed using the GUM estimation approach. However, the GUM analysis might not sufficiently account for the nonlinearity of the multiparametric data analysis of the binary measurements.^49^ Therefore, the uncertainties were additionally estimated using a Monte Carlo (MC) method. For the GUM approach, an extended formulation for the combined uncertainty of the absolute adsorbed amount according to eq 19 was used. The respective standard uncertainties of the 11 different contributions are listed in Table 3 and we used a coverage factor of *k* = 2 to calculate the combined expanded uncertainties. This table also shows an example calculation of the GUM approach for the absolute adsorbed amount of CH_4_ at a pressure of 1 MPa and 333 K with mixture 1, including the individual contributions of the 11 input values. The uncertainty of the equilibrium composition is inherently considered in the individual contributions from *W*_12_, *p*, *T*, *V*_sk_, and ρ_EOS_, which were used to calculate *y*_eq_.
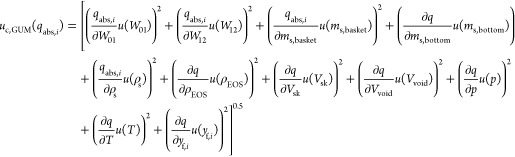
19

For the Monte Carlo uncertainty estimation, the values used in the data analysis were considered with their standard uncertainties as shown in Table 3. Therefore, all 11 input values were randomly varied by applying a normal distribution to their standard uncertainty. The size of the random sampling was 1000 runs. The standard deviation of the absolute adsorbed amount in the 1000 runs was then used as the combined uncertainty *u*_c,MC_, which was converted to an extended combined uncertainty considering *k* = 2.

### 2.4 Equilibrium Modeling

#### 2.4.1 Unary Gases

To model the equilibrium data of the unary gases on RM 8850, we used the simplified statistical isotherm (SSI) model developed by Ruthven^32^ for zeolitic materials. The model describes adsorption as a pore-filling process, occurring only in the cages of the zeolite. The zeolite has a constant cage volume ν_c_ [Å^3^], yielding a maximum number of adsorbate molecules ω per cage. The adsorbed molecules have an effective volume β [Å^3^] and interactions between adsorbate molecules are neglected. Adsorbate–adsorbent interactions are considered by the temperature-dependent Henry’s law affinity parameter *K* [molecules/cage/MPa]. The absolute adsorbed amount of molecules per cage *n*_abs,c_ [molecules/cage] is defined as
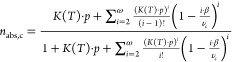
20

The Henry’s law affinity parameter is given by an Arrhenius expression considering the temperature-independent Henry’s law affinity parameter *K*_0_ [molecules/cage/MPa], the molar gas constant *R* [kJ/mol/K], and the adsorption enthalpy Δ*E*_ads_ [kJ/mol]:
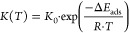
21

The absolute adsorbed amount per cage is converted to the absolute adsorbed amount of mole per mass *q*_abs_ [mmol/g] considering the cage volume ν_c_, the micropore volume ν_mi_, and the Avogadro constant *N*_A_ [molecules/mmol]:
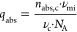
22

The maximum integer number of adsorbed molecules ω per cage was estimated by considering the molecular van der Waals covolume β_vdW_ of the molecules, i.e., ω = ν_c_/β_vdW_, for which β_vdW,CO2_ = 70.9 Å^3^ and β_vdW,CH4_ = 71.0 Å^3^ were used.^50^ The volume of a single cage was taken as *v*_c_ = 958.2 Å^3^.^51^ The model was fitted to the experimentally determined absolute amount adsorbed *q*_abs_ (*p*,*T*) by adjusting *K*_0_, β, and Δ*E*_ads_ as fitting parameters for both gases individually. The root-mean-square deviation (RMSD) was used as objective function considering the number of data points *N* and minimized by using the built-in MATLAB function *fmincon*:
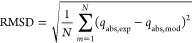
23

#### 2.4.2 Binary Gases

The binary formulation of the SSI model developed by Ruthven^34^ was used to model the binary adsorption data. The model assumes a partition function for a cage filling with *i* molecules of CO_2_ (component A) and *j* molecules of CH_4_ (component B), carried out over all values for *i* and *j*, which satisfy the restriction *iβ*_A_ + *jβ*_B_ ≤ ν_c_. The mixture composition is considered as the partial pressure of the two components, i.e., *p*_A_ and *p*_B_. For component A, the absolute amount of molecules adsorbed per cage *n*_abs,c,A_ [mmol/g] can be described according to eq 24. The absolute adsorbed amount per mass *q*_abs,A_ can be calculated as for the unary case according to eq 22.
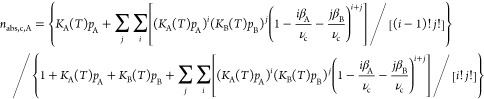
24

We used two different approaches to obtain the parameters for the binary model. In the first approach (referred to as predictive binary modeling, PBM), we used the SSI parametrization obtained from the unary adsorption data and applied it to the binary model in a purely predictive fashion. In the second approach (referred to as weighted binary modeling, WBM), we fitted *K*_0,A_, *K*_0,B_, β_A_, β_B_, Δ*E*_ads,A_, and Δ*E*_ads,B_ to the full set of unary and binary data. Because the binary data has comparably large measurement uncertainties, the combined expanded uncertainties *U*_c_ of the data were considered as a weighting factor in the objective function
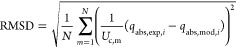
25

#### 2.4.3 Uncertainty Estimation of Model Parameters

To estimate the uncertainty of the model parameters, the Monte Carlo method for uncertainty propagation was used by considering the combined expanded uncertainties of the experimental data. The sample size was 1000 individual fits. In each fit, each experimental data point was randomly varied in accordance with a normal distribution of the individual combined expanded uncertainties. The standard deviation of the 1000 sample fits was then used as the uncertainty of the individual parameters.

## 3 Results and Discussion

### 3.1 Unary Adsorption

**Figure 2:**
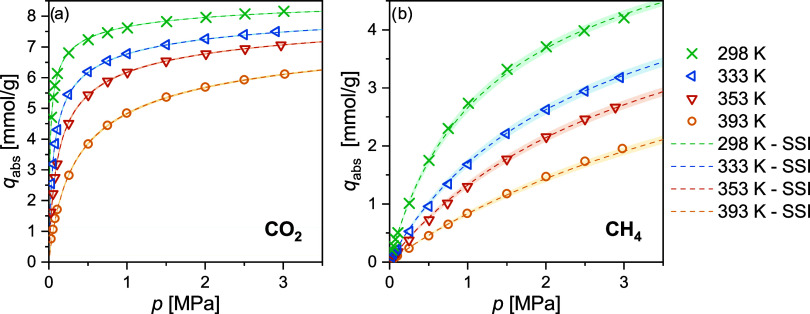
Absolute adsorption loading *q*_abs_ of pure CO_2_ (a) and CH_4_ (b) on RM 8850 at four different temperatures and pressures up to 3 MPa and the results of the SSI modeling. Shaded areas show the uncertainty from the model. Experimental uncertainties are not shown as the error bars are smaller than the symbols.

The absolute adsorption isotherms of pure CO_2_ and CH_4_ on RM 8850 at four different temperatures are shown in Figure 2. The data are additionally reported in Tables 5 and 6, together with the estimated uncertainties in the amount adsorbed. The CO_2_ adsorption isotherms show a steep increase already at low pressures, so that, e.g., 7.615 mmol/g is adsorbed at 0.1 MPa and 298 K. In comparison, the CH_4_ adsorption isotherms show a less steep initial increase, yielding much lower adsorption loadings (e.g., 0.494 mmol/g at 0.1 MPa and 298 K). The data shown in Figure 2 have been measured on the pelletized samples. A comparison of these data against independent measurements carried out on RM 8850 powder with CO_2_ and CH_4_ is shown in the SI S4. We observe excellent agreement between the two data sets. Specifically, for CO_2_, the results are in excellent agreement with the data from Azzan et al.^51^ on RM 8850 powder, which were obtained at 298, 333, 353, and 393 K. For CH_4_, our results at 298 K are within the reported uncertainty of the reference excess adsorption isotherm model by the NIST interlaboratory study.^14^ It can therefore be concluded that the shaping of RM 8850 does not influence the adsorption equilibria.

Figure 2 also shows the adsorption isotherms described by the SSI model, which was fitted to the experimental data. The SSI model parameters are given in Table 4, including their uncertainties. The model provides an excellent description of the CO_2_ and CH_4_ adsorption at all temperatures and over the entire pressure range. Similarly good fits of the SSI model were reported by Azzan et al.^51^ for CO_2_, N_2_, and H_2_ adsorption on a RM 8850 powder sample. The shaded areas in Figure 2 represent the uncertainty bounds associated with the model fits. Since the experimental uncertainties of the CH_4_ measurements are higher compared to CO_2_ while the adsorption loading is less, the CH_4_ model parameters possess significantly higher uncertainties.

**Table 4:** Model Parameters for the Simplified Statistical Isotherm (SSI) Model Are Determined by Fitting the Parameters to the Unary CO_2_ and CH_4_ Data^a^

	ω	*K*_0_ × 10^4^	–Δ*E*_ads_	β
	molec/cage	molec/cage/MPa	kJ/mol	Å^3^
CO_2_	14	13.229 (0.202)	33.675 (0.045)	57.542 (0.027)
CH_4_	14	97.202 (4.128)	16.933 (0.125)	58.316 (0.500)

a The values in parentheses give the uncertainty of the parameters.

The physical meaning of the obtained parameters is discussed in the following. As the molecular van der Waals covolumes β_vdW_ for CO_2_ and CH_4_ are similar (see Section 2.4), both maximum integer numbers for the saturation capacity ω = 14 molec/cage are the same. This saturation capacity corresponds to *q*_abs_^ω^ = 8.7 mmol/g, as obtained upon application of eq 22. Upon comparing this value to the measured amount of CO_2_ adsorbed at 3 MPa and 298 K, we conclude that the micropores of the adsorbent are close to saturation (*q*_abs_/*q*_abs_^ω^ ≈ 0.91). On the contrary, *q*_abs_/*q*_abs_^ω^ ≈ 0.46 when CH_4_ is used, meaning that much higher pressures are needed to reach saturation at 298 K. These differences are due to the relative distance of the experimental temperature to the critical temperature of the adsorbate, i.e., *T*_c,CO2_ = 304.15 K > 298 K vs *T*_c,CH4_ = 190.56 K ≪ 298 K. The fitted values for β are similar for both gases (approximately 58 Å^3^) and smaller than the molecular van der Waals covolumes (approximately 71 Å^3^), which can be ascribed to compressibility effects at high pressure. Henry’s law parameters *K*_0_ and Δ*E*_ads_ represent the temperature dependency of the adsorption isotherm. The temperature dependence of the adsorption loading and the emerging plateauing at higher pressure for the CO_2_ isotherms are represented well by the parameters. Azzan et al.^51^ have also shown that the SSI model provides an accurate description of these parameters for the Henry’s law region by comparing them in van’t Hoff plots with parameters obtained directly from independent low-pressure isotherm measurements.

### 3.2 Binary Adsorption

**Figure 3:**
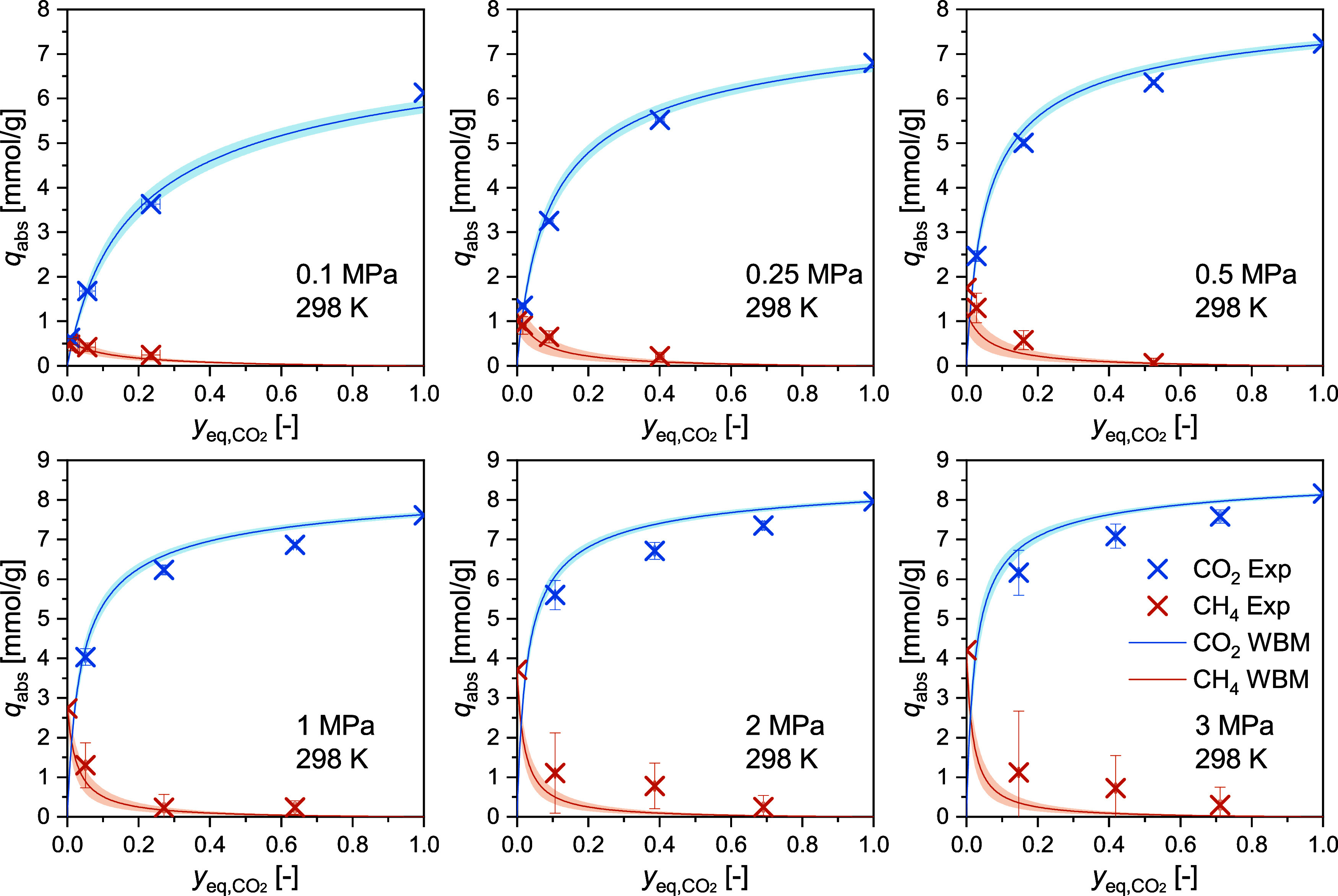
Competitive absolute adsorbed loadings of CO_2_ and CH_4_ (see eq 16) against the equilibrium CO_2_ concentration of the gas phase (see eq 12) on RM 8850 at a temperature of *T* = 298 K and pressures up to 3 MPa. In each plot, three binary adsorption data points are shown for each gas species as well as the unary data points at *y*_eq,CO2_ = 1 (pure CO_2_) and *y*_eq,CO2_ = 0 (pure CH_4_). Results of the weighted binary modeling (WBM) of the simplified statistical isotherm model are shown as solid lines. Shaded areas show the uncertainty from the model.

**Figure 4:**
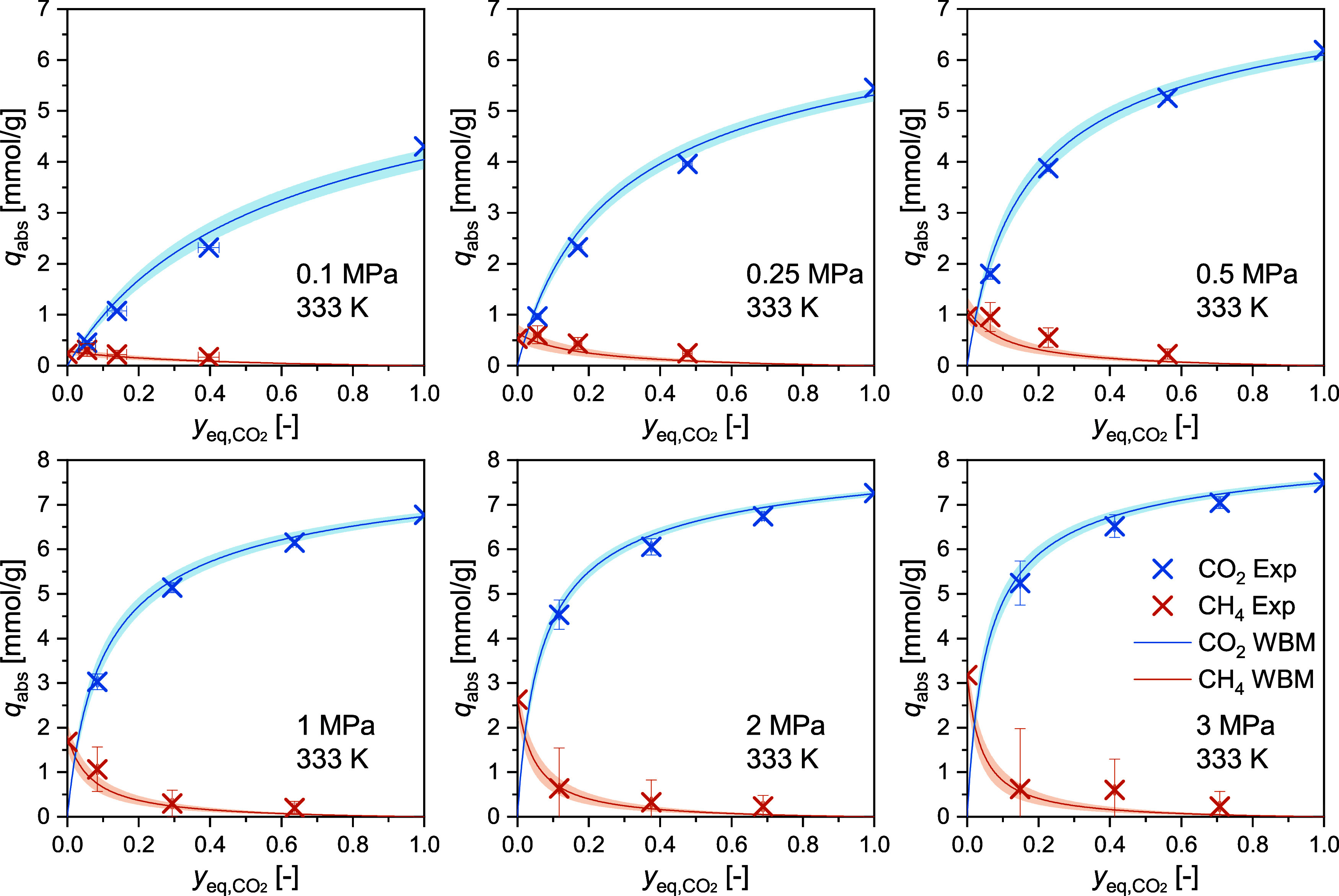
Competitive absolute adsorbed loadings of CO_2_ and CH_4_ (see eq 16) against the equilibrium CO_2_ concentration of the gas phase (see eq 12) on RM 8850 at a temperature of *T* = 333 K and pressures up to 3 MPa. In each plot, three binary adsorption data points are shown for each gas species as well as the unary data points at *y*_eq,CO2_ = 1 (pure CO_2_) and *y*_eq,CO2_ = 0 (pure CH_4_). Results of the weighted binary modeling (WBM) of the simplified statistical isotherm model are shown as solid lines. Shaded areas show the uncertainty from the model.

**Figure 5:**
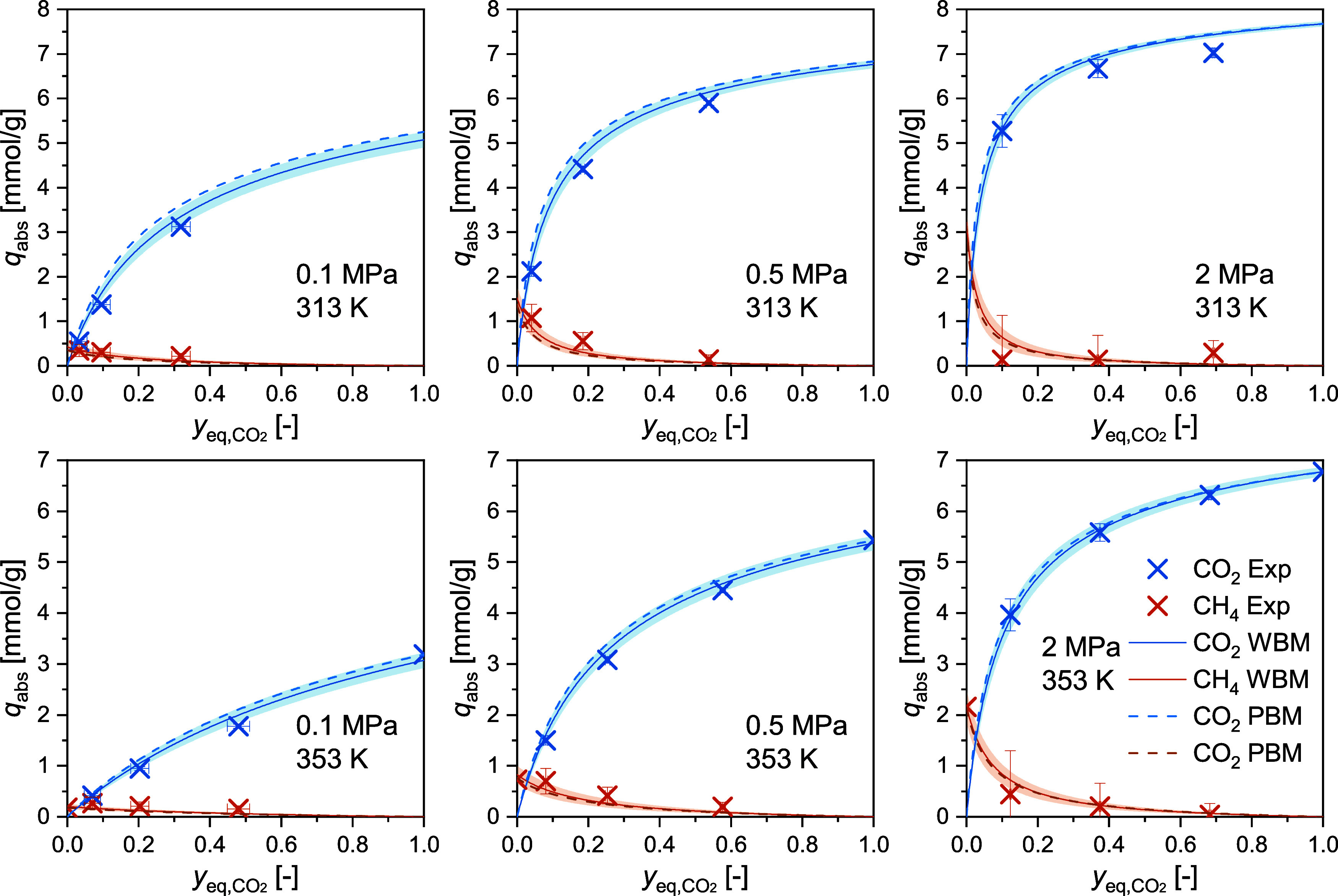
Competitive absolute adsorbed loadings of CO_2_ and CH_4_ (see eq 16) against the equilibrium CO_2_ concentration of the gas phase (see eq 12) on RM 8850 at *T* = 313 and 353 K at pressures up to 2 MPa. In each plot, three binary adsorption data points are shown for each gas species. In the plots at *T* = 353 K, the unary data points at *y*_eq,CO2_ = 1 (pure CO_2_) and *y*_eq,CO2_ = 0 (pure CH_4_). The data is compared with the predictive binary modeling (PBM) and the weighted binary modeling (WBM). Shaded areas show the uncertainty from the WBM model.

The binary adsorption measurements were conducted with three CO_2_+CH_4_ mixtures (see feed gas compositions in Table 2) at pressures from 0.1 to 3 MPa at *T* = 298 and 333 K and from 0.1 to 2 MPa at *T* = 313 and 353 K. The measurements were conducted isothermally with increasing pressure for each gas mixture. The competitive absolute adsorbed loadings for CO_2_ and CH_4_ are listed in Table 7 and the individual isothermal measurements are separated by horizontal lines. The corresponding competitive excess adsorbed loadings are listed in SI S6. The binary adsorption isotherms are plotted at selected pressures as a function of the equilibrium CO_2_ gas phase mole fraction in Figure 3 (*T* = 298 K), in Figure 4 (*T* = 333 K), and in Figure 5 (*T* = 313 and 353 K). The unary values (cf. Tables 5 and 6) for pure CO_2_ (*y*_CO2_ = 1) and CH_4_ (*y*_CO2_ = 0) are also shown in these figures. As the static gravimetric method relies on a composition shift due to the selective adsorption in the closed cell, the CO_2_ mole fraction in equilibrium varies significantly from the mole fraction of the feed gas. This effect is particularly pronounced at low pressures (<0.5 MPa), where the equilibrium points are gathering toward the left-hand side of the graphs.

**Table 5:** Excess and Absolute Amount Adsorbed of CO_2_ on RM 8850 with Expanded Uncertainties *U*(*q*) considering a Coverage Factor of *k* = 2 at Temperature *T* and Pressure *p*

*T*	*p*	*q*_ex_	*q*_abs_	*U* (*q*_ex_)	*U* (*q*_abs_)
[K]	[MPa]	[mmol/g]	[mmol/g]	[mmol/g]	[mmol/g]
298.05	3.008	7.639	8.160	0.022	0.022
298.05	2.509	7.652	8.070	0.022	0.022
298.05	2.010	7.637	7.961	0.022	0.022
298.05	1.505	7.588	7.823	0.022	0.022
298.05	1.005	7.463	7.615	0.022	0.022
298.05	0.750	7.347	7.459	0.022	0.022
298.05	0.504	7.160	7.234	0.022	0.022
298.05	0.254	6.764	6.801	0.022	0.022
298.05	0.110	6.117	6.133	0.021	0.021
298.05	0.074	5.730	5.740	0.021	0.021
298.05	0.053	5.348	5.355	0.021	0.021
298.06	0.033	4.706	4.710	0.020	0.020
333.05	2.908	7.076	7.492	0.022	0.022
333.05	2.499	7.047	7.399	0.022	0.022
333.01	2.005	6.983	7.260	0.022	0.022
333.09	1.502	6.862	7.065	0.022	0.022
333.05	1.003	6.642	6.775	0.021	0.021
333.05	0.750	6.446	6.545	0.021	0.021
333.06	0.504	6.127	6.193	0.021	0.021
333.08	0.253	5.418	5.451	0.021	0.021
333.06	0.111	4.290	4.304	0.020	0.020
333.07	0.083	3.840	3.851	0.019	0.019
333.05	0.053	3.186	3.193	0.019	0.019
333.05	0.033	2.543	2.548	0.019	0.019
353.05	2.972	6.676	7.068	0.021	0.021
353.05	2.510	6.619	6.945	0.021	0.021
353.05	2.010	6.521	6.778	0.021	0.021
353.04	1.501	6.354	6.543	0.021	0.021
353.04	1.007	6.059	6.184	0.021	0.021
353.04	0.752	5.799	5.892	0.021	0.021
353.05	0.504	5.378	5.439	0.020	0.021
353.05	0.255	4.486	4.517	0.020	0.020
353.05	0.107	3.181	3.194	0.019	0.019
353.03	0.078	2.746	2.756	0.019	0.019
353.05	0.052	2.222	2.229	0.018	0.018
353.03	0.027	1.623	1.626	0.018	0.018
393.04	3.018	5.764	6.111	0.021	0.021
393.00	2.501	5.642	5.926	0.021	0.021
393.00	2.002	5.467	5.693	0.020	0.021
392.95	1.502	5.195	5.362	0.020	0.020
392.94	0.997	4.733	4.843	0.020	0.020
392.95	0.752	4.362	4.445	0.020	0.020
393.01	0.503	3.786	3.840	0.019	0.019
393.05	0.258	2.791	2.819	0.019	0.019
393.02	0.105	1.699	1.711	0.018	0.018
392.95	0.079	1.408	1.417	0.018	0.018
392.95	0.052	1.058	1.064	0.017	0.017
392.83	0.029	0.755	0.759	0.017	0.017

**Table 6:** Excess and Absolute Amount Adsorbed of CH_4_ on RM 8850 with Expanded Uncertainties *U*(*q*) considering a Coverage Factor of *k* = 2 at Temperature *T* and Pressure *p*

*T*	*p*	*q*_ex_	*q*_abs_	*U* (*q*_ex_)	*U* (*q*_abs_)
[K]	[MPa]	[mmol/g]	[mmol/g]	[mmol/g]	[mmol/g]
298.05	2.999	3.753	4.209	0.048	0.048
298.14	2.490	3.610	3.985	0.048	0.048
298.10	2.005	3.414	3.713	0.048	0.048
298.05	1.505	3.101	3.323	0.048	0.048
298.15	1.007	2.590	2.737	0.048	0.048
298.10	0.751	2.195	2.305	0.047	0.047
298.07	0.503	1.679	1.752	0.047	0.047
298.15	0.251	0.976	1.012	0.046	0.047
298.07	0.105	0.494	0.509	0.046	0.046
298.06	0.075	0.384	0.395	0.046	0.046
298.05	0.046	0.276	0.283	0.046	0.046
298.05	0.027	0.203	0.207	0.046	0.046
332.93	2.950	2.784	3.176	0.048	0.048
333.05	2.502	2.612	2.942	0.047	0.048
333.11	2.006	2.363	2.627	0.047	0.047
333.09	1.492	2.014	2.209	0.047	0.047
333.05	1.004	1.552	1.683	0.047	0.047
333.11	0.752	1.247	1.344	0.047	0.047
333.15	0.504	0.894	0.959	0.046	0.046
333.07	0.259	0.493	0.527	0.046	0.046
333.05	0.107	0.218	0.231	0.046	0.046
333.05	0.082	0.191	0.202	0.046	0.046
333.03	0.051	0.130	0.137	0.046	0.046
333.05	0.026	0.079	0.082	0.046	0.046
352.93	2.890	2.306	2.665	0.047	0.047
353.05	2.501	2.154	2.464	0.047	0.047
353.05	2.004	1.911	2.158	0.047	0.047
353.05	1.500	1.589	1.773	0.047	0.047
353.05	0.998	1.179	1.301	0.047	0.047
353.05	0.741	0.926	1.016	0.046	0.046
353.05	0.506	0.665	0.726	0.046	0.046
352.95	0.249	0.348	0.379	0.046	0.046
352.91	0.109	0.162	0.175	0.046	0.046
352.99	0.067	0.102	0.111	0.046	0.046
353.05	0.051	0.080	0.086	0.046	0.046
353.05	0.025	0.044	0.047	0.046	0.046
392.93	2.980	1.624	1.953	0.047	0.047
392.92	2.501	1.458	1.734	0.047	0.047
393.20	2.003	1.252	1.472	0.047	0.047
392.98	1.500	1.012	1.177	0.046	0.046
392.87	0.995	0.727	0.836	0.046	0.046
392.92	0.750	0.568	0.650	0.046	0.046
392.95	0.502	0.396	0.451	0.046	0.046
392.92	0.250	0.211	0.238	0.046	0.046
392.88	0.101	0.094	0.106	0.046	0.046
392.98	0.079	0.077	0.086	0.046	0.046
392.99	0.049	0.052	0.058	0.046	0.046
393.04	0.030	0.036	0.039	0.046	0.046

Table 7 also includes the extended combined uncertainties (*k* = 2) estimated using the GUM and the MC approach for both gases. The MC calculations lead to higher uncertainty values compared to the GUM approach, since the former covers better the nonlinearity of the data analysis and allows for a more rigorous sampling. Accordingly, in Figures 3 to 5, the error bars represent the uncertainty values obtained using the MC approach. The higher measurement uncertainties for CH_4_ can be explained by the lower molar mass and density of CH_4_. We note that the measurements conducted with mixture 1 (cf. Table 2) show high uncertainties for CH_4_ – particularly at elevated pressures. As indicated by the exemplary analysis of the individual contributions of the 11 input values using the GUM approach (see Table 3), the dominant contribution to the combined uncertainty comes from the mixture composition. Mixture 1 possesses the highest uncertainty in composition as stated by the supplier, reflected in the larger uncertainties for these measurements.

**Table 7:** Absolute Amount Adsorbed of CO_2_ and CH_4_ from Binary Mixtures with Equilibrium CO_2_ Mole Fractions *y*_eq,CO2_ on RM 8850 with Expanded Uncertainties *U*_c,GUM_(*q*) Determined by GUM and Standard Uncertainties *U*_c,MC_(*q*) Determined by MC^a^

*T*	*p*	*y*_eq,CO2_	*q*_abs,CO2_	*q*_abs,CH4_	*U*_c,GUM_ (*q*_CO2_)	*U*_c,GUM_ (*q*_CH4_)	*U*_c,MC_ (*q*_CO2_)	*U*_c,MC_ (*q*_CH4_)
[K]	[MPa]	[–]	[mmol/g]	[mmol/g]	[mmol/g]	[mmol/g]	[mmol/g]	[mmol/g]
298.05	0.101	0.008	0.629	0.474	0.031	0.090	0.046	0.135
298.05	0.251	0.016	1.346	0.905	0.044	0.124	0.069	0.197
298.05	0.512	0.028	2.464	1.302	0.072	0.199	0.120	0.333
298.05	1.006	0.051	4.035	1.303	0.122	0.336	0.207	0.568
298.05	1.993	0.107	5.600	1.105	0.217	0.597	0.369	1.012
298.05	3.005	0.146	6.162	1.117	0.315	0.866	0.567	1.553
								
298.05	0.110	0.056	1.678	0.420	0.030	0.073	0.041	0.092
298.05	0.244	0.090	3.248	0.649	0.036	0.093	0.055	0.136
298.05	0.500	0.160	5.009	0.577	0.050	0.131	0.081	0.213
297.95	0.999	0.271	6.233	0.232	0.073	0.197	0.124	0.333
298.05	2.003	0.386	6.713	0.779	0.124	0.339	0.211	0.576
298.05	2.998	0.419	7.088	0.724	0.179	0.490	0.302	0.827
								
298.05	0.106	0.234	3.631	0.242	0.030	0.062	0.043	0.067
298.05	0.260	0.400	5.521	0.218	0.032	0.069	0.049	0.084
298.05	0.508	0.525	6.362	0.062	0.036	0.080	0.056	0.108
297.99	1.010	0.639	6.866	0.237	0.045	0.109	0.072	0.166
298.05	1.996	0.691	7.354	0.242	0.069	0.181	0.116	0.299
298.05	2.997	0.712	7.582	0.295	0.101	0.273	0.170	0.453
								
312.95	0.116	0.033	0.539	0.346	0.030	0.087	0.044	0.133
312.95	0.498	0.041	2.121	1.075	0.065	0.181	0.111	0.309
312.99	2.005	0.100	5.270	0.134	0.201	0.552	0.365	0.997
								
312.95	0.108	0.096	1.377	0.303	0.028	0.070	0.040	0.090
312.95	0.494	0.184	4.414	0.553	0.046	0.121	0.073	0.191
312.95	2.005	0.369	6.673	0.132	0.116	0.317	0.203	0.554
								
313.05	0.113	0.319	3.124	0.216	0.029	0.060	0.041	0.064
313.01	0.503	0.538	5.899	0.140	0.034	0.076	0.054	0.105
313.05	2.016	0.692	7.025	0.293	0.065	0.169	0.109	0.276
								
333.03	0.112	0.055	0.448	0.308	0.029	0.084	0.042	0.123
333.04	0.248	0.056	0.966	0.608	0.038	0.108	0.060	0.173
333.05	0.502	0.064	1.799	0.955	0.061	0.168	0.102	0.282
333.05	1.008	0.084	3.028	1.065	0.105	0.289	0.178	0.500
333.05	2.014	0.117	4.535	0.635	0.189	0.518	0.330	0.908
333.05	3.043	0.148	5.243	0.623	0.275	0.756	0.495	1.355
								
333.05	0.103	0.139	1.072	0.217	0.027	0.066	0.037	0.084
333.05	0.248	0.169	2.326	0.435	0.032	0.081	0.048	0.116
333.05	0.527	0.227	3.877	0.551	0.044	0.116	0.072	0.190
333.05	1.014	0.294	5.140	0.297	0.065	0.174	0.112	0.297
333.05	1.999	0.375	6.055	0.324	0.108	0.294	0.184	0.501
333.01	2.986	0.413	6.518	0.601	0.154	0.421	0.255	0.695
								
333.01	0.108	0.396	2.319	0.168	0.028	0.057	0.039	0.063
333.05	0.247	0.476	3.961	0.244	0.029	0.061	0.044	0.072
333.01	0.506	0.562	5.255	0.227	0.033	0.072	0.053	0.099
333.05	1.014	0.637	6.150	0.198	0.040	0.096	0.065	0.146
332.97	2.011	0.689	6.742	0.229	0.059	0.154	0.099	0.250
333.05	2.998	0.708	7.047	0.229	0.083	0.221	0.130	0.341
								
352.95	0.121	0.070	0.412	0.267	0.028	0.082	0.042	0.120
352.99	0.500	0.080	1.501	0.699	0.055	0.154	0.090	0.251
352.95	1.989	0.123	3.966	0.442	0.172	0.474	0.315	0.859
								
353.01	0.115	0.203	0.947	0.205	0.026	0.064	0.039	0.088
352.95	0.498	0.253	3.083	0.418	0.040	0.103	0.064	0.166
352.95	1.980	0.374	5.585	0.201	0.100	0.271	0.171	0.458
								
353.05	0.109	0.480	1.778	0.153	0.027	0.054	0.039	0.063
352.95	0.494	0.577	4.449	0.195	0.031	0.067	0.048	0.090
352.95	1.999	0.682	6.320	0.034	0.055	0.141	0.090	0.225

a In both cases, a coverage factor of *k* = 2 is considered. The individual isothermal measurements are separated by the horizontal lines.

Such rigorous uncertainty analyses (with 11 input values) as we carried out for the binary adsorption investigations are rarely found in the literature for measurements with a magnetic suspension balance, so it is difficult to compare the uncertainty values with other studies. However, one study worth mentioning is that of Yang et al.,^23^ in which the adsorption of a N_2_+CO_2_ and a N_2_+CH_4_ mixture on commercial sodium molecular sieve type Y beads was investigated. They assessed the uncertainty using the GUM approach and reported uncertainties for the adsorption equilibria from 0.02 up to 0.4 mmol/g. Most of our reported uncertainties from the GUM approach agree with this range. However, Yang et al. assumed a much less conservative estimate for the uncertainty in the gas mixture composition than we did, leading to their lower uncertainty values in general.

In Figures 3 to 5, the experimental results are also compared with the results of the weighted binary model fitting (WBM) of the SSI model. The obtained model parameters are reported in Table 8 alongside their uncertainties. Across all four temperatures, the WBM-SSI model describes the results well, and in most cases, the experimental data is represented within their uncertainty. At 313, 333, and 353 K, the model shows low deviations at all investigated pressures. Higher deviations are observed at 298 K and pressures from 1 MPa upward. As 298 K was the lowest investigated temperature, it might be that the temperature dependency is not fully covered by the model at these high pressures.

**Table 8:** Model Parameters Were Obtained by the Weighted Binary Model Fitting (WBM) for the SSI Model^a^

	*K*_0_ × 10^4^	–Δ*E*_ads_	β
	molec/cage/MPa	kJ/mol	Å^3^
CO_2_	24.657 (0.621)	31.585 (0.072)	56.846 (0.046)
CH_4_	58.858 (5.570)	18.806 (0.287)	67.075 (1.125)

a The values in parentheses give the uncertainty of the parameters.

To assess the applicability of the predictive binary modeling (PBM), PBM-SSI and WBM-SSI are compared with the experimental data at 313 and 353 K in Figure 5; comparisons for the other temperatures are shown in the SI S5. In general, the WBM shows lower deviations to the experimental data than the PBM – as expected, given that the WBM was fitted to both the binary and unary data. However, we acknowledge that the differences between the two approaches are rather minor and become negligible at higher pressures. The ability to predict the mixture adsorption data from the pure component isotherms bears important practical implications–reducing substantially the experimental effort. Further work is recommended to assess whether this conclusion can be drawn for other gas mixtures and zeolite types.

In the following, we compare the model parameters of the WBM approach (Table 8) with the PBM approach (Table 4). The values of the effective volume β of CO_2_ obtained using the two approaches are very similar (approximately 57 Å^3^), while for CH_4_ the WBM approach yields a larger value of β (approximately 67 Å^3^) than the PBM approach (approximately 58 Å^3^). The fact that during competitive adsorption CO_2_ behaves similarly as during unary adsorption is likely the result that the adsorbed phase remains much richer in CO_2_ than CH_4_ at all conditions investigated here. As a result, the adsorbed CH_4_ molecules experience a weaker compressibility effect, yielding a larger value of β. We note, however, that this value remains smaller than the theoretical van der Waals covolume. For the adsorption enthalpy, similar values were obtained for both adsorptives from the two approaches. The obtained Henry’s law parameter *K*_0_ shows the largest differences between the two approaches, with the WBM approach yielding a slightly better-described pressure dependency (see Figure 5). Due to the higher experimental uncertainties of the binary measurements, the uncertainty values of the WBM parameters are two to three times higher than the uncertainties of the PBM parameters.

**Figure 6:**
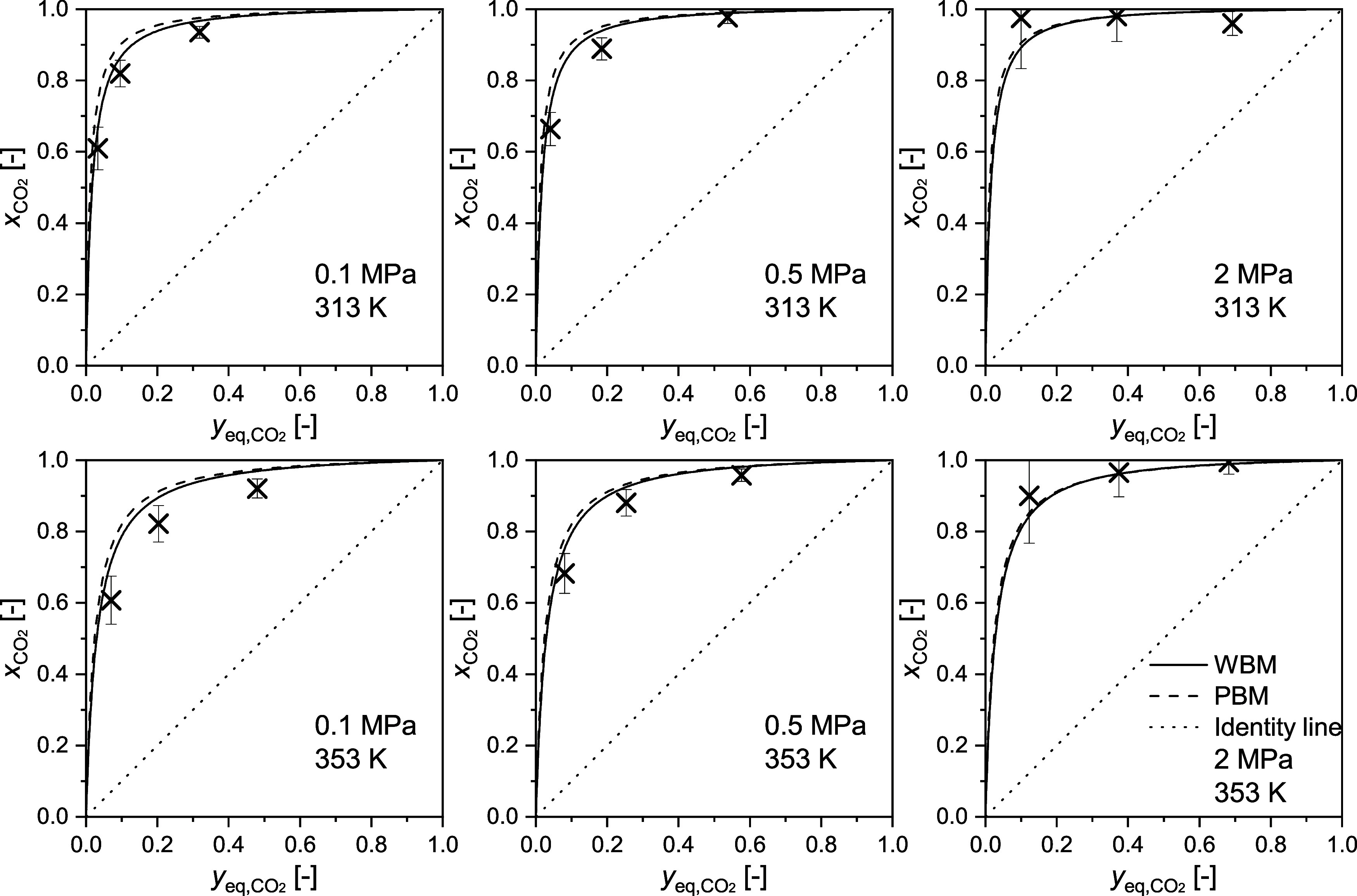
Comparison of the predictive binary modeling (PBM) and the weighted binary modeling (WBM) with the CO_2_ mole fraction of the adsorbed phase on RM 8850 at *T* = 313 and 353 K at pressures up to 2 MPa. The identity line represents the case of a nonselective adsorption process.

To assess the selectivity of RM 8850, the CO_2_ mole fraction of the adsorbed phase *x*_CO2_ is shown versus the mole fraction of the gas phase *y*_CO2_ in Figure 6 for 313 and 353 K; the plots for the other temperatures are shown in the SI S5. The dotted identity line represents the case of a nonselective adsorption process, i.e., meaning that no separation would occur. As the experimental values significantly deviate from the nonselective case, RM 8850 shows, as expected, a highly selective affinity for CO_2_ adsorption over CH_4_ adsorption. WBM and PBM in general describe this selective process well, but the WBM again shows a slightly better performance.

## 4 Conclusions

We report unary excess and absolute adsorbed loadings of CO_2_ and CH_4_ at temperatures from 298 to 393 K and pressures up to 3 MPa as well as competitive absolute adsorbed loadings of binary CO_2_+CH_4_ mixtures at temperatures from 298 to 353 K and pressure up to 3 MPa on NIST reference zeolite RM 8850 pellets. A comparison of the unary CO_2_ and CH_4_ adsorption with literature data on RM 8850 powder shows that the unary equilibria properties of the pellets and powder agree well. The binary data set is the first reported data on any of the NIST reference materials and shows a strong selectivity of RM 8850 for CO_2_ adsorption over CH_4_. We modeled the unary adsorption data using the simplified statistical isotherm model for zeolites developed by Ruthven.^32^ We used the obtained parametrization in the extended binary formulation of the model to predict the binary adsorption of CO_2_+CH_4_ mixtures and compared it with a parametrization obtained by fitting it to the full set of unary and binary data. Both approaches represent well the experimental data, though the latter describes the binary data set with lower deviation. Given that binary adsorption measurements are very time-intensive, our study suggests that the predictive model may be sufficient in some cases. In addition, the reported data can be used by the adsorption community to compare and validate binary adsorption experiments. Going forward, binary adsorption of further gas mixtures should be investigated on reference materials.

## Data Availability

The unary and binary adsorption data is available in adsorption information format (AIF) files and in a spreadsheet in the Supporting Information.

## Supporting Information Available

Unary and binary adsorption data (ZIP)
Information on: the determination of the feed gas mixture composition; the determination of the total void volume of the magnetic suspension balance; the experimental properties for the unary and binary adsorption data analysis; comparisons of the predictive and fitted binary modeling approach; and binary excess adsorption data (PDF)
