# Supplementary material for: Binary Adsorption Equilibria of Three CO2+CH4 Mixtures on NIST Reference Zeolite Y (RM 8850) at Temperatures from 298 to 353 K and Pressures up to 3 MPa
Source: J Chem Eng Data. 2024 Oct 3;69(11):4216–29. doi: 10.1021/acs.jced.4c00358 (PMC11571112; doi:10.1021/acs.jced.4c00358)
Supplement: Supplementary file 2 — je4c00358_si_002.pdf [file je4c00358_si_002.pdf]

Supporting Information (SI) for "Binary adsorption equilibria of three CO_2+CH_4 mixtures on NIST reference zeolite Y (RM8850) at temperatures from 298 to 353 K and pressures up to 3 MPa"

Carsten Wedler[1*], Alvaro Ferre[2], Hassan Azzan[1], David Danaci[1,3,4], Camille Petit[1], Ronny Pini[1]

[1]*Department of Chemical Engineering, Imperial College London, SW7 2AZ, London, United Kingdom*

[2]*Laboratory of Chemical Process Engineering, Technical University of Munich, 94315 Straubing, Germany*

[3]*The Sargent Centre for Process Systems Engineering, Imperial College London, SW7 2AZ, London, United Kingdom*

[4]*I-X Centre for AI in Science, Imperial College London, W12 0BZ, London, United Kingdom*

*Corresponding author: c.wedler@imperial.ac.uk

S1. Determination of the feed gas mixture composition

The gas mixture composition of the pre-mixed cylinders used in the competitive adsorption experiments was investigated using the calibrated sinker of the magnetic suspension balance coupled with calculations from an appropriate equation of state. The configuration of the balance for these investigations was as described for the binary adsorption measurements, apart from removing all traces of the adsorbent from the system. The measurement cell and all included piping were evacuated for 1 h, followed by filling the gas mixture under study at a temperature of 298 K. We set the first measurement point at 1 MPa and waited for a pressure-temperature equilibrium for about 1 h. We alternated between measurement positions 0, 1, and 2, recorded the weighing values W_1 and W_2 for about 30 min, and averaged the values over this period. Afterwards, the pressure was increased in 0.5 MPa steps up to 3 MPa.

We used Eqs. (3) and (4) from the main manuscript to determine the density of the gas mixture. The experimental density, temperature, and pressure were then used as input for the GERG-

2008 equation of state [1] (as implemented in REFPROP 10.0 [2]) to calculate the mole fractions of the gas mixtures according to Eq. (12) from the main manuscript. The calculated mole fractions for the five pressure steps (1, 1.5, 2, 2.5, and 3 MPa) were averaged and the averaged values are reported for the three gas mixtures in Table 2 of the main manuscript.

S2. Determination of the total void volume of the magnetic suspension balance

We determined the total void volume V_{void} of the magnetic suspension balance by conducting expansion experiments with an inert gas. Therefore, an additional vessel was connected to the gravimetric instrument as shown in Figure S 1.

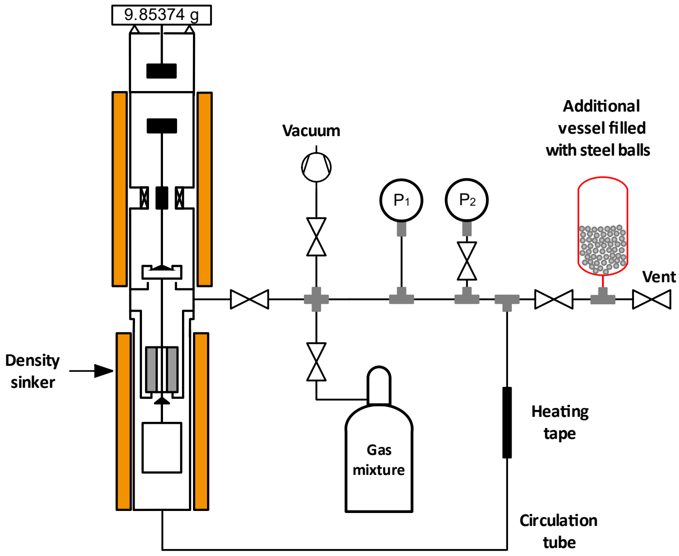

Figure S 1. The gravimetric adsorption system is extended by an additional vessel (marked red) to conduct expansion experiments.

With this modification, two different sets of experiments were conducted: (A) measurements with the empty vessel, and (B) with the vessel filled with 493 stainless steel balls with a diameter of 8 mm. The total volume of the balls was calculated to yield V_{ball} = 132.16 cm³. Before each measurement, the system, including the vessel, was evacuated for 1 h. We then closed the valve between the gravimetric instrument and the vessel, and filled the instrument with argon up to a pressure of 3 MPa. Argon was chosen as it is an inert gas with a comparably

high density. After 1 h of equilibration, the density (index 1) of the gas was determined in the same way as described in S1. Then, we opened the valve to the evacuated vessel, waited for equilibrium for about 1 h, and repeated the measurement of the gas density (index 2). Given a constant mass of gas during the experiment, a mass balance can be established for each of the two cases (A and B), which can be reduced according to Eqs. (S1) and (S2). Solving them according to Eq. (S3), allows us to calculate V_{void}. Each set of experiments was conducted 4 times, leading to 4 slightly different values for V_{void} shown in Table S 1. The average value of these 4 experiments was used in the data analysis of the main manuscript. For the uncertainty analysis of the binary adsorption measurements, the standard uncertainty of this approach was conservatively estimated to be 1 cm^3.

$$\frac{\rho_{\text{A1}}}{\rho_{\text{A2}}} = \frac{V_{\text{void}} + V_{\text{vessel}}}{V_{\text{void}}} \tag{S1}$$

$$\frac{\rho_{\text{B1}}}{\rho_{\text{B2}}} = \frac{V_{\text{void}} + V_{\text{vessel}} - V_{\text{balls}}}{V_{\text{void}}} \tag{S2}$$

$$V_{\text{void}} = \frac{V_{\text{balls}}}{\frac{\rho_{\text{A1}}}{\rho_{\text{A2}}} - \frac{\rho_{\text{B1}}}{\rho_{\text{B2}}}} \tag{S3}$$

Table S 1. Values for the total void volume V_{void} of the magnetic suspension balance were obtained from four different experiments and the average value was used for the data analysis.

	Exp 1	Exp 2	Exp 3	Exp 4	$\bar{V}_{\text{void}}$
V_{void} [cm^3]	175.312	175.646	175.308	175.650	175.48

S3. Experimental properties for the unary and binary adsorption data analysis

Table S 2 shows the values considered in the data analysis of the unary and binary experiments.

Table S 2. Volumes, masses, weighing values, and skeletal density considered in the data analysis of the unary and binary experiments.

m_{met} [g]	7.70461
V_{met} [cm^3]	1.4195
V_{sk} [cm^3]	4.3640

V_{void} [cm³]	175.48
v_{mi} [cm³/g]	0.358
ρ_s [g/cm³]	2.523
Unary experiments	
$m_{s,basket}$ [g]	0.79740
Binary experiments	
$m_{s,basket}$ [g]	1.30230
$W_{s,basket}$ [g]	1.36990
$W_{s,bottom}$ [g]	4.03480

S4. Comparison of unary adsorption on pellets and powder

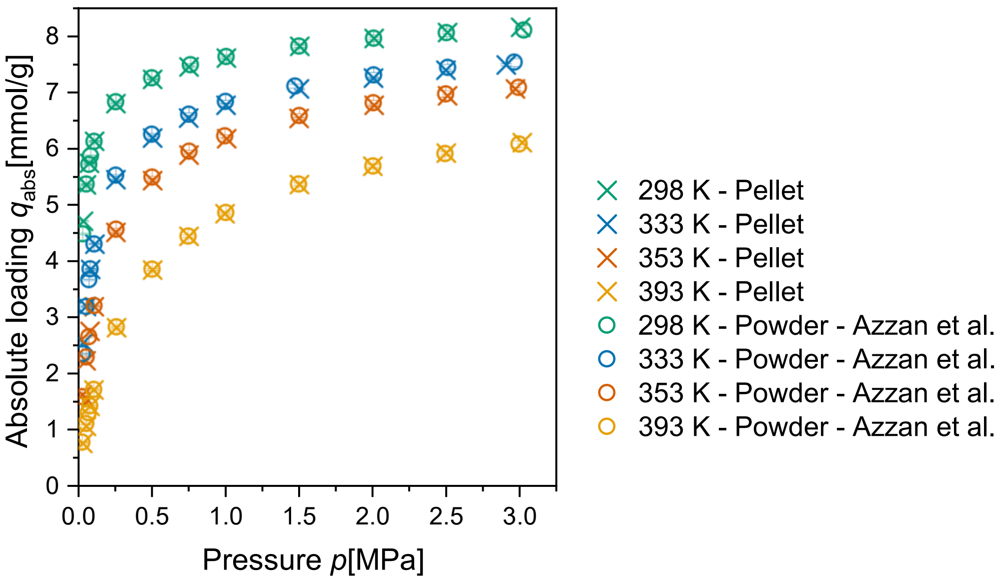

Figure S 2. Comparison of absolute adsorption of CO_2 on RM8850 pellets (this work) and RM 8850 powder measured by Azzan et al. [3] at 4 different temperatures.

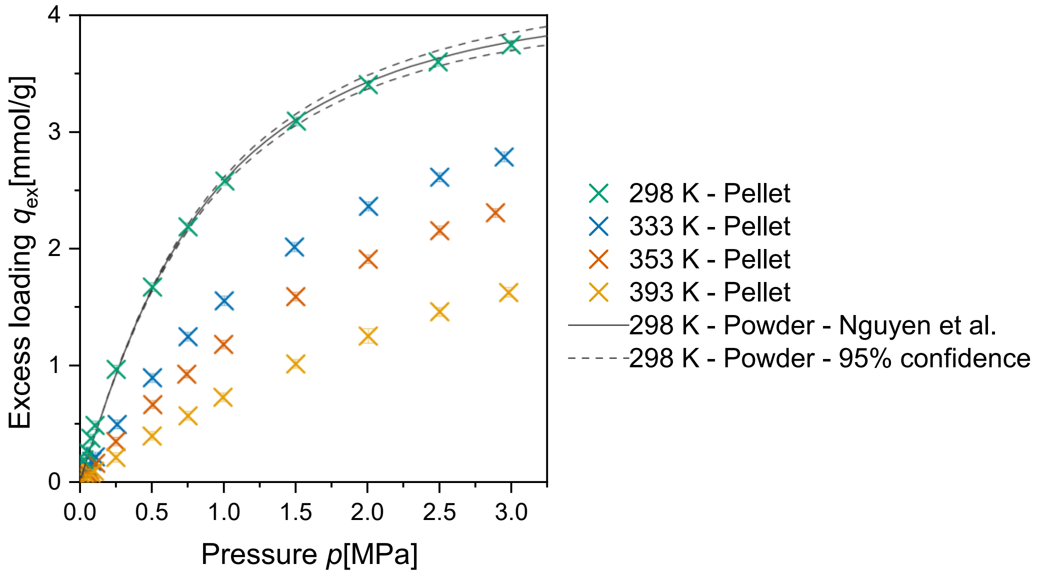

Figure S 3. Comparison of excess adsorption of CH_4 on RM8850 pellets (this work) and the reference excess isotherm model on RM 8850 powder reported by Nguyen et al. [4] at 298 K.

S5. Comparison of the predictive and fitted binary modelling approach

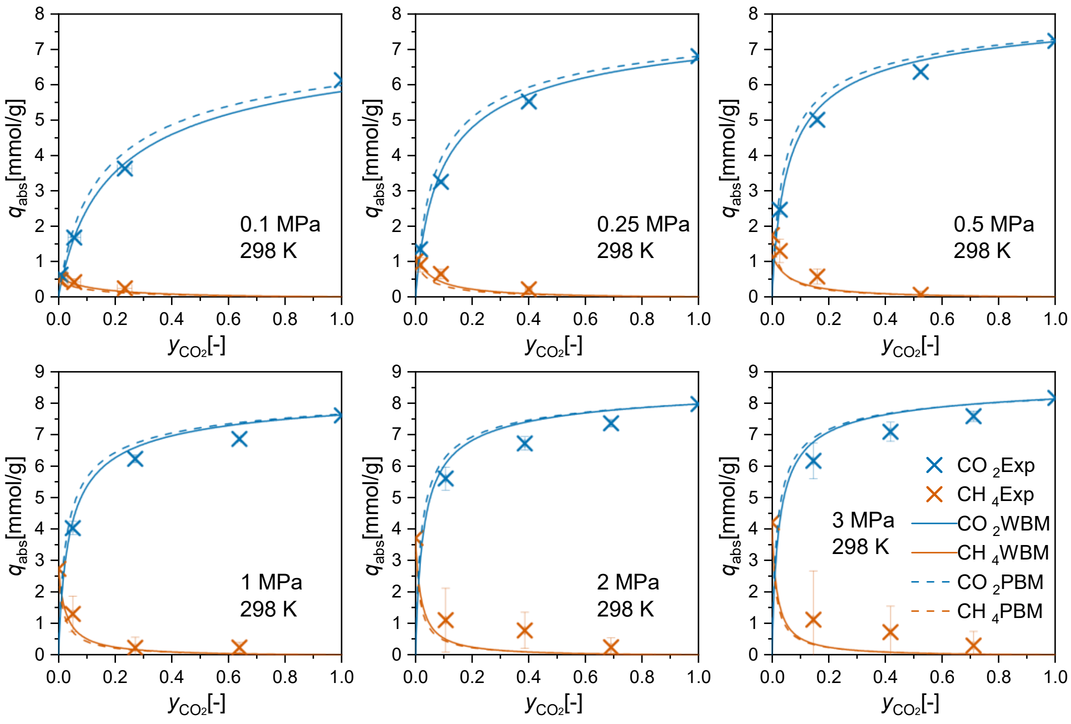

Figure S 4. Comparison of the predictive binary modelling (PBM) and the weighted binary modelling (WBM) with the experimental competitive adsorption loadings for CO_2 and CH_4 on RM 8850 at 298 K at pressures up to 3 MPa.

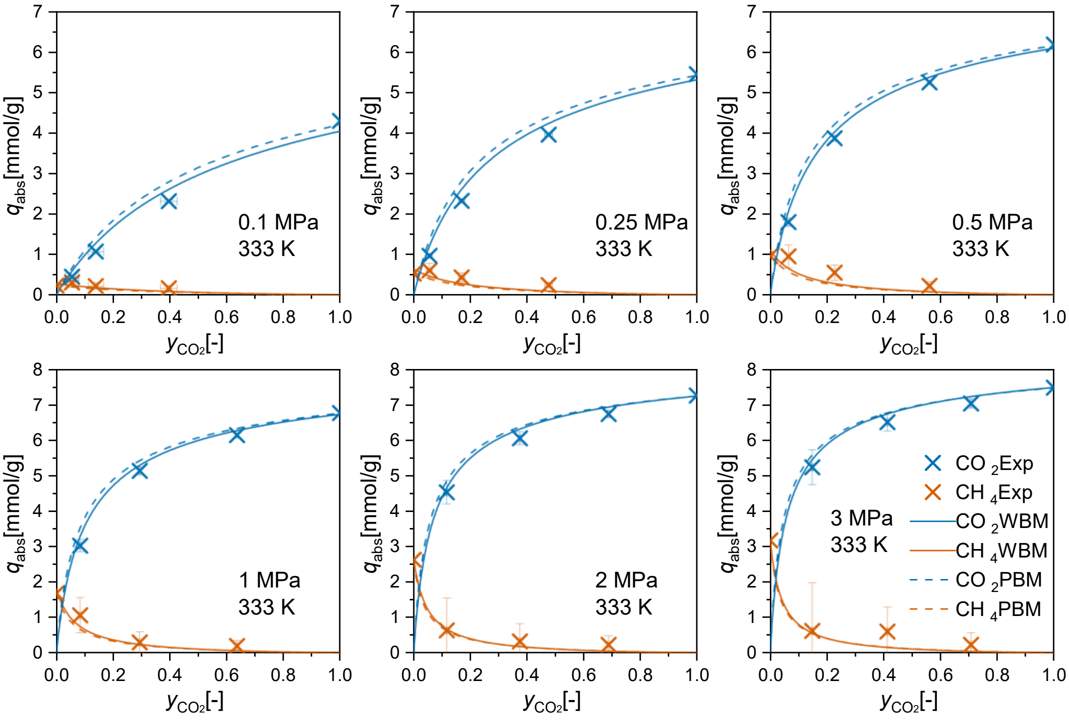

Figure S 5. Comparison of the predictive binary modelling (PBM) and the weighted binary modelling (WBM) with the experimental competitive adsorption loadings for CO_2 and CH_4 on RM 8850 at 333 K at pressures up to 3 MPa.

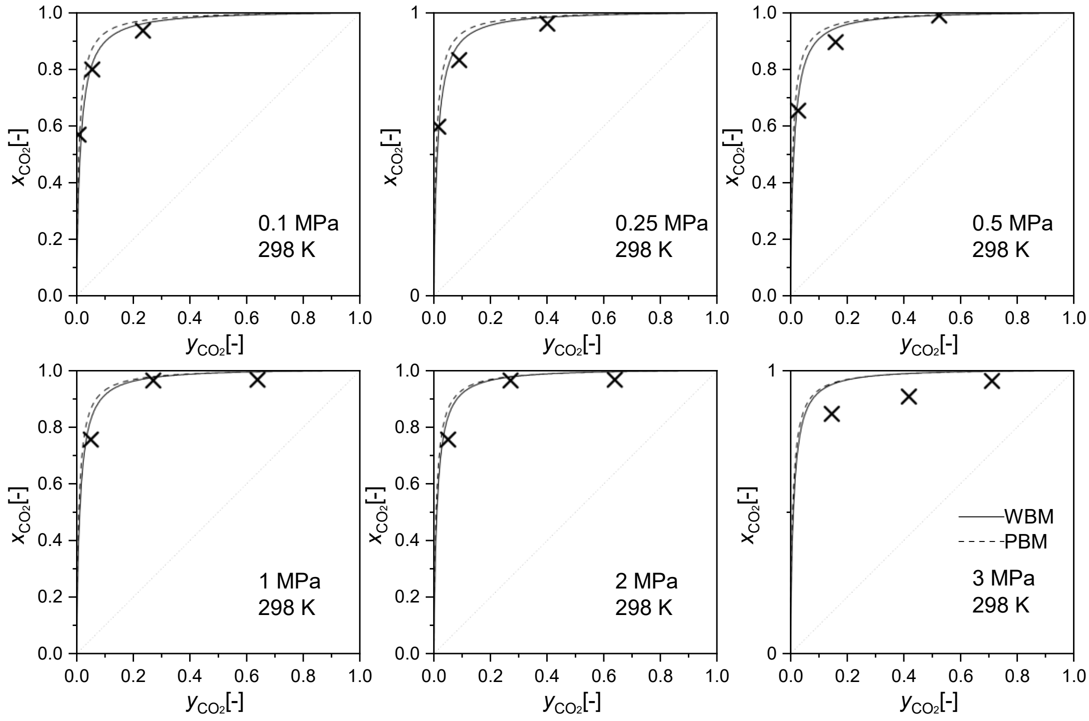

Figure S 6. Comparison of the predictive binary modelling (PBM) and the weighted binary modelling (WBM) with the CO_2 mole fraction of the adsorbed phase on RM 8850 at 298 K at pressures up to 3 MPa.

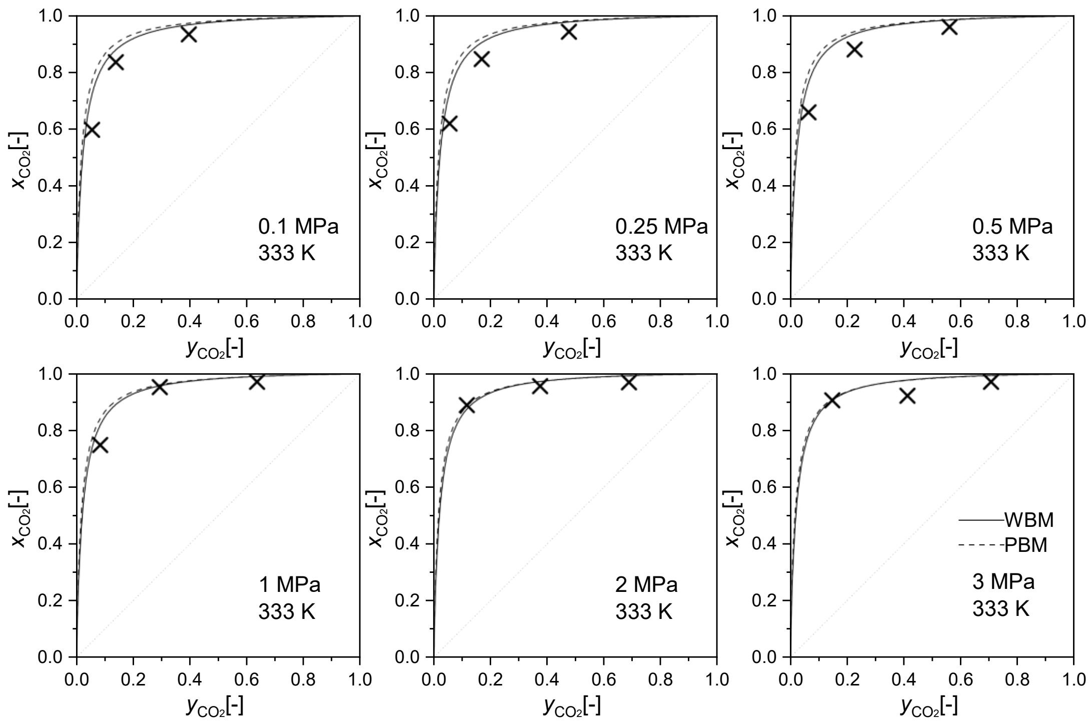

Figure S 7. Comparison of the predictive binary modelling (PBM) and the weighted binary modelling (WBM) with the CO_2 mole fraction of the adsorbed phase on RM 8850 at 333 K at pressures up to 3 MPa.

S6. Binary excess adsorption data

The excess adsorbed loadings for the binary experiments $q_{\mathrm{ex},i}$ was calculated according to Eq. (S4) and is listed in **Table S 3**.

$$q_{\mathrm{ex},i} = \frac{m_{\mathrm{ex},i}}{m_{\mathrm{s,basket}} + m_{\mathrm{s,bottom}}} \cdot \frac{1}{M_i} \quad \text{(S4)}$$

Table S 3. Excess amount adsorbed of CO_2 and CH_4 from binary mixtures with an equilibrium CO_2 mole fractions $y_{\mathrm{eq,CO2}}$ on RM 8850.

T	p	$y_{\mathrm{eq,CO2}}$	$q_{\mathrm{ex,CO2}}$	$q_{\mathrm{ex,CH4}}$
[K]	[MPa]	[-]	[mmol/g]	[mmol/g]
298.05	0.101	0.008	0.628	0.458
298.05	0.251	0.016	1.346	0.869
298.05	0.512	0.028	2.462	1.229
298.05	1.006	0.051	4.027	1.162
298.05	1.993	0.107	5.568	0.838
298.05	3.005	0.146	6.094	0.723
298.05	0.110	0.056	1.677	0.405
298.05	0.244	0.090	3.245	0.617
298.05	0.500	0.160	4.997	0.515

T	p	$y_{eq,CO2}$	$q_{ex,CO2}$	$q_{ex,CH4}$
[K]	[MPa]	[-]	[mmol/g]	[mmol/g]
297.95	0.999	0.271	6.192	0.124
298.05	2.003	0.386	6.595	0.591
298.05	2.998	0.419	6.890	0.449
298.05	0.106	0.234	3.628	0.230
298.05	0.260	0.400	5.506	0.196
298.05	0.508	0.525	6.323	0.027
297.99	1.010	0.639	6.769	0.183
298.05	1.996	0.691	7.138	0.146
298.05	2.997	0.712	7.232	0.153
312.95	0.116	0.033	0.538	0.330
312.95	0.498	0.041	2.118	1.009
312.99	2.005	0.100	5.242	-0.122
312.95	0.108	0.096	1.375	0.289
312.95	0.494	0.184	4.402	0.497
312.95	2.005	0.369	6.567	-0.050
313.05	0.113	0.319	3.119	0.205
313.01	0.503	0.538	5.861	0.108
313.05	2.016	0.692	6.820	0.201
333.03	0.112	0.055	0.447	0.294
333.04	0.248	0.056	0.965	0.577
333.05	0.502	0.064	1.795	0.894
333.05	1.008	0.084	3.017	0.944
333.05	2.014	0.117	4.504	0.399
333.05	3.043	0.148	5.182	0.275
333.05	0.103	0.139	1.071	0.206
333.05	0.248	0.169	2.320	0.408
333.05	0.527	0.227	3.862	0.498
333.05	1.014	0.294	5.101	0.203
333.05	1.999	0.375	5.954	0.157
333.01	2.986	0.413	6.350	0.361
333.01	0.108	0.396	2.313	0.159
333.05	0.247	0.476	3.946	0.227
333.01	0.506	0.562	5.218	0.198
333.05	1.014	0.637	6.064	0.149
332.97	2.011	0.689	6.554	0.144
333.05	2.998	0.708	6.750	0.106
352.95	0.121	0.070	0.411	0.253
352.99	0.500	0.080	1.496	0.642
352.95	1.989	0.123	3.936	0.225
353.01	0.115	0.203	0.944	0.194
352.95	0.498	0.253	3.067	0.372
352.95	1.980	0.374	5.492	0.046
353.05	0.109	0.480	1.772	0.146
352.95	0.494	0.577	4.414	0.169
352.95	1.999	0.682	6.147	-0.046

References

[1] Kunz O, Wagner W. The GERG-2008 Wide-Range Equation of State for Natural Gases and Other Mixtures: An Expansion of GERG-2004. J Chem Eng Data 2012;57(11):3032-91.

[2] Huber ML, Lemmon EW, Bell IH, McLinden MO. The NIST REFPROP Database for Highly Accurate Properties of Industrially Important Fluids. Ind Eng Chem Res 2022;61(42):15449-72.

[3] Azzan H, Danaci D, Petit C, Pini R. Unary Adsorption Equilibria of Hydrogen, Nitrogen, and Carbon Dioxide on Y-Type Zeolites at Temperatures from 298 to 393 K and at Pressures up to 3 MPa. J Chem Eng Data 2023;68(12):3512-24.

[4] Nguyen HGT, Sims CM, Toman B, Horn J, van Zee RD, Thommes M, Ahmad R, Denayer JFM, Baron GV, Napolitano E, Bielewski M, Mangano E, Brandani S, Broom DP, Benham MJ, Dailly A, Dreisbach F, Edubilli S, Gumma S, Möllmer J, Lange M, Tian M, Mays TJ, Shigeoka T, Yamakita S, Hakuman M, Nakada Y, Nakai K, Hwang J, Pini R, Jiang H, Ebner AD, Nicholson MA, Ritter JA, Farrando-Pérez J, Cuadrado-Collados C, Silvestre-Albero J, Tampaxis C, Steriotis T, Řimnáčová D, Švábová M, Vorokhta M, Wang H, Bovens E, Heymans N, De Weireld G. A reference high-pressure CH4 adsorption isotherm for zeolite Y: results of an interlaboratory study. Adsorption 2020;26(8):1253-66.
